# CGRP release in an experimental human trigeminal pain model

**DOI:** 10.1177/03331024211017250

**Published:** 2021-06-20

**Authors:** Achim Frese, Oliver Summ, Stefan Evers

**Affiliations:** 1 Faculty of Medicine, University of Münster, Germany; 2 Akademie für Manuelle Medizin, Münster, Germany; 3 Department of Neurology and Research Center of Neurosensory Science, Carl von Ossietzky University Oldenburg, Oldenburg, Germany; 4 Department of Neurology, Lindenbrunn Hospital, Coppenbrügge, Germany

**Keywords:** CGRP, capsaicin, headache, trigemino-autonomic symptoms

## Abstract

**Background:**

Migraine and trigemino-autonomic cephalalgia attacks are associated with an increase of α-calcitonin-gene related peptide levels in the ipsilateral jugular vein. It is however unknown whether trigeminal pain stimulation in healthy subjects without headache disorders also induces increase of calcitonin-gene related peptide levels.

**Findings:**

We measured α-calcitonin-gene related peptide levels in eight healthy subjects after subcutaneous injection of capsaicin in the forehead and in the mandibular region and after injection of sodium chloride in the forehead. We observed a significant increase of α-calcitonin-gene related peptide level only after injection of capsaicin in the forehead (i.e. first trigeminal branch). We also observed trigemino-autonomic activation (lacrimation, rhinorrhea etc.) only after injection of capsaicin in the forehead.

**Conclusion:**

Increase of α-calcitonin-gene related peptide levels do not only occur in primary headache attacks but also after experimental trigeminal pain of the first branch. This finding suggests that α-calcitonin-gene related peptide elevation is, at least an additional, unspecific effect of first trigeminal branch stimulation following pain activation and not a specific mechanism of idiopathic headache disorders.

## Introduction

Calcitonin-gene related peptide (CGRP) is regarded today as the most important and central molecule/neuropeptide in migraine pathophysiology. This is based, initially, on clinical studies on elevated α-CGRP levels in trigeminal pain disorders such as trigeminal neuralgia, migraine, cluster headache, and paroxysmal hemicrania. It has been shown that α-CGRP levels are increased after stimulation of the trigeminal ganglion in patients with trigeminal neuralgia (1), in the migraine attack (2), in the cluster headache attack (3) and even, as baseline level, in chronic migraine (4). However, it is not yet completely clear whether the release of α-CGRP in these disorders starts before the onset of pain or whether this release follows the pain. In some secondary headache disorders such as cervicogenic headache, no release of α-CGRP during the pain phase was noted (5).

We aimed to study the release of α-CGRP in an experimental trigeminal pain model, in particular we were interested in differences between the first trigeminal branch pain and other trigeminal pain.

## Methods

We enrolled eight healthy subjects (3 female, 5 male; mean age 28 +/− 4 years; all right-handed) without any history of a primary headache disorder, only infrequent episodic tension-type headache was allowed. Even in first degree relatives, no migraine history was allowed. Subjects had to be without any medication including painkillers in the week before. The subjects were examined three times. They received:

- an injection of capsaicin subcutaneously in the right forehead
- an injection of capsaicin subcutaneously in the right mandibular region
- an injection of physiological NaCl solution subcutaneously in the right forehead

The order of the injections was randomised for every patient. The patient was not told whether he/she received capsaicin or NaCl. The study protocol was approved by the local ethics committee of the University of Münster. All subjects gave written informed consent following a detailed explanation of the procedure. 0.05 ml of capsaicin 0.01% (or NaCl solution) were injected subcutaneously into the forehead/mandibular region of healthy volunteers without any history of headache. The appearance of autonomic symptoms was carefully observed and documented. Subjects rated their pain every 10 seconds within the first 2 minutes, then every minute for 13 minutes on a visual analogue scale from 0 (no pain) to 10 (maximal pain).

The blood samples were always taken between 10 am and 4 pm, the first sample was taken as baseline, the second within 10 minutes when the patient noted maximal pain intensity and then after 15 minutes. Subjects were in an upright position. A small ‘butterfly’-cannula (21G) was inserted into the ipsilateral and the contralateral external jugular vein and the antecubital vein on the nondominant side. Thus, we obtained three blood samples per injection. Blood (9 ml) was collected into tubes prepared with 15% ethyl-enediaminetetraacetate and immediately decanted into tubes prepared with aprotinin (Trasylol®, Bayer, Leverkusen, Germany). The tubes were cooled on ice and centrifuged at 2,000 g for 15 minutes. Plasma for α-CGRP was aliquoted into polypropylene tubes for storage at −80°C until analysis. Samples were coded and all determinations were performed blinded. Plasma α-CGRP concentrations were measured with a fully evaluated radioimmunoassay for human α-CGRP, as described previously. In brief, human α-CGRP was obtained from Peninsula Laboratories Europe (St. Helens, UK). Standards were prepared in 0.1 mol/l phosphate buffer (pH 7.5) containing 0.1% bovine serum albumin, 0.01% sodium azide, and 20 kIU/ml aprotinin (Trasylol®). The tracer was prepared by iodination of [Tyr0] α-CGRP25-37 (Multiple Peptide Systems, San Diego, CA, USA) using the Iodo Gen method (Pierce, Rockford, IL, USA) and purified by high-performance liquid chromatography on a Merck-Hitachi system (E. Merck, Darmstadt, Germany). The antibody used was raised in rabbits against human α-CGRP and is specific for the C-terminal segment of α-CGRP. It was used in a final dilution of 1: 600,000. The assay was performed at 4°C as a nonequilibrium assay incubating sample (100 µl) and antibody (200 µl) for 4 days prior to 2 days of incubation with tracer. Free and antibody-bound tracer were separated using a solid-phase separation system (SAC-CEL; IDS, Boldon, UK). The detection limit of the assay was 5 pmol/l. The intra-assay coefficient of variation was 2%, and the inter-assay coefficient of variation was 7%. The non-specific binding was 3% to 4%. Measurements were done in triplicate.

Primary endpoint for the data analysis was the increase of α-CGRP between injection and maximal pain. Results are presented as arithmetic mean +/- standard deviation. For comparing α-CGRP-values, the nonparametric Friedman-test was used with Wilcoxon-test as post hoc test. All statistical analyses were performed using SPSS version 15.0 (SPSS, Chicago, IL, USA). The level of significance was set at P< 0.05.

## Results

The pain intensity was rated between 7 and 10 by all subjects within one minute after capsaicin injection when injected both in the forehead and in the mandibular region. Pain intensity after NaCl injection was up to 2. All subjects except one showed trigemino-autonomic symptoms only after injection of capsaicin in the forehead (lacrimation = 6; [incomplete] Horner’s syndrome = 4; conjunctival injection = 3; nasal congestion = 1) for up to 15 minutes.

**Figure 1.**
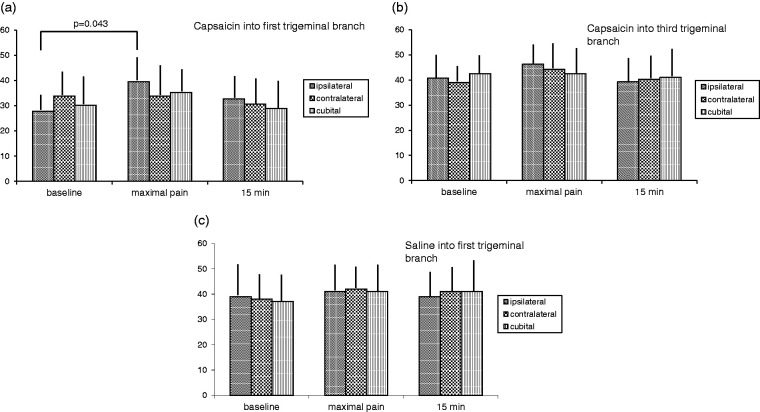
a to c: α-CGRP levels in pmol/l at baseline, at pain maximum and after 15 minutes for all three different blood samples presented as arithmetic mean and standard deviation. 1 a: injection of capsaicin into the first trigeminal branch 1 b: injection of capsaicin into the third trigeminal branch 1 c: injection of saline into the first trigeminal branch

At baseline, no significant differences of the α-CGRP level were observed for all three conditions. After injection of capsaicin in the forehead, the α-CGRP level in the ipsilateral jugular vein increased from 28 +/- 5 to 40 +/- 7 pmol/l (p=0.043), whereas no significant increase was observed in the contralateral jugular vein and in the cubital vein. After injection of NaCl in the mandibular region and in the forehead, no significant changes of the α-CGRP level in all veins was observed, respectively. The changes of α-CRGP are presented in Figure 1a to 1c.

## Discussion

Our main finding is a significant α-CGRP release into the blood of the ipsilateral external jugular vein when evoking severe trigeminal pain in the first trigeminal branch but not in the third trigeminal branch. This finding is supported by a placebo control and suggests that the α-CGRP release in headache disorders is mainly due to first branch trigeminal pain activation and not a specific mechanism of only idiopathic headache disorders. This mechanism is most likely caused by activation of the sphenopalatine ganglion reflex which can be regarded as an unspecific reflex to pain. Furthermore, the data suggest that the pain intensity is more important than the pain duration for α-CGRP release which is triggered by pain induction and not by specific mechanisms of idiopathic headache disorders. This would mean that in idiopathic headache disorders, α-CGRP release is probably not the first mechanism to induce the pain.

In models of newborn animals, stimulation by capsaicin induced α-CGRP release in several tissues (6). Also, in other animal models, stimulation of the dura mater led to a release of α-CGRP into the cerebral vessels (7).

In an experimental human model, direct stimulation of nerve fibres in the dental pulp (i.e. second trigeminal branch) resulted in an α-CGRP release however with very high inter-subject variability (8). In another model, oral intake of capsaicin induced α-CGRP release into the saliva in a dose dependent manner (9). Also, intradermal stimulation by an inflammatory soup including capsaicin induced a release of α-CGRP into jugular vein blood (10). Activation of α-CGRP release was also demonstrated by capsaicin induced stimulation of the nasal mucosa (11); however, this was shown in cluster headache patients and not in healthy control, so it could not be concluded from that study whether the effect of capsaicin is really unspecific.

The question remains whether the acute trigeminal pain is a trigger for α-CGRP release also in idiopathic headache disorders. This problem cannot be answered by our experimental study, we can only conclude that severe pain in the first trigeminal branch is a trigger for α-CGRP release in healthy subjects. It might be that this is an unspecific effect. It might also be that in idiopathic headache disorders the pain also initiates α-CGRP release but that this release again increases pain intensity leading to a vicious circle. It should be noted in this context that the α-CGRP level increases by about 100% in migraine or even more in cluster headache as compared to baseline whereas in our study the increase was only about 40%.

There are some limitations to be discussed with respect to this study. First, we did not investigate the second branch which could show different results. Sometimes, in migraine (so-called facial migraine) and in trigemino-autonomic cephalalgias, the second branch is even more affected than the first branch. Also, it would be interesting to observe α-CGRP activation in different phases of a migraine cycle. Second, the subject sample was quite small and very homogenous. It is, however, difficult to find volunteers for such painful studies who never had migraine themselves or in their family; this was also the reason why more male subjects were included than expected from migraine epidemiology. However, we are not aware of sex differences in α-CGRP levels. We first aimed to look into the applicability of the study procedure in order to study other pain locations later. From our results, it seems that this method of pain and α-CGRP measurements is possible and thus presents a model to study the involvement of α-CGRP in human pain processing.

## Key findings

- Capsaicin injected in the first trigeminal branch induces ipsilateral α-CGRP release in the external jugular vein and activates trigemino-autonomic symptoms.
- Capsaicin injected in the third trigeminal branch does not impact α-CGRP release.
